# Lost in translation: why genomic breakthroughs are not reaching patients

**DOI:** 10.1136/bmjonc-2026-001107

**Published:** 2026-05-17

**Authors:** Andrew Calcino, Matt Field

**Affiliations:** 1 Centre for Tropical Bioinformatics and Molecular Biology; College of Science and Engineering, James Cook University, Cairns, Queensland, Australia; 2 Garvan Institute of Medical Research, Darlinghurst, New South Wales, Australia

**Keywords:** Genetic markers, Health economics, Autoimmunity, Biostatistics

## Abstract

Despite revolutionary advances in genomic technologies, a persistent disconnect exists between research discoveries and clinical implementation. This translational gap stems from misaligned incentive and funding structures: researchers prioritise publications over clinical uptake, with funding ending at proof-of-concept; clinicians face time constraints and integration challenges; regulators struggle with rapidly evolving technologies and genomics-specific complexities including variant classification and data governance. We propose that dedicated translational medicine centres are essential to bridge this divide. These centres require multidisciplinary teams spanning clinician-scientists, regulatory affairs specialists, health economists, biostatisticians and bioinformaticians, providing end-to-end support from feasibility assessment through to regulatory approval. Success requires government investment, explicit health equity assessments and measuring achievement through clinical uptake rather than traditional academic metrics.

 The journey from recognising patient variability to modern molecular precision medicine spans millennia. Early physicians, including Hippocrates, observed variable treatment responses in patients, proposing therapies to balance individual humoral composition. William Osler further championed this individualised approach, stating: “the good physician treats the disease, the great physician treats the patient who has the disease”.^1^ The molecular era started with Archibald Garrod’s work on inborn errors of metabolism, proposing that biochemical differences account for variable disease susceptibility.^2^

It is now feasible to interrogate individual patient genetic landscapes at unprecedented resolution, with emerging single-cell technologies able to elucidate genetic variation from the whole genomes of individual cells.^3^ Despite these advances, a persistent and widening disconnect exists between the pace of genomic discovery and the translation of these discoveries into clinical practice. While media reports routinely describe scientific breakthroughs presumably ready for patient care, translating discoveries into practice is laborious and costly. At the core of the problem is a misalignment of both incentive and funding structures between research funding bodies, health systems and medical regulators. In this editorial, we examine the structural barriers impeding this translation and propose a model for dedicated translational medicine centres to help bridge this gap.

In the standard modern laboratory, academic success is measured by publications and grants, with clinical uptake secondary. Funding typically ends at proof-of-concept with researchers, having little exposure to the nuances of regulatory processes and clinical trials.^4^ Results from controlled research environments often fail to scale clinically due to factors including variable reagent and equipment quality, small sample sizes, poor reproducibility due to incomplete methodological documentation and lack of validation across representative populations.^5^

For clinicians, time constraints and workloads limit capacity to test new technologies. Training on interpreting new molecular tests is limited, and integrating new tests into existing information technology systems and health system workflows is challenging. Discoveries often lack prospective clinical trial evidence, creating liability concerns, while robust health economic data and evidence of clear clinical actionability are often lacking.^6^

For regulators, technology now outpaces regulatory guidelines (eg, artifical intelligence/machine leaning-based medicine), making appropriate evidence standards difficult to define. Specific to genomics, issues including changes to variant classification, secondary/incidental findings and balancing data sharing with privacy protection compound the problem.^7 8^ Economic silo-based thinking represents an additional challenge. Health systems must constantly compete for funding with other social and political priorities, and even where new approaches have been demonstrated to be cost-effective at a system level, innovations are unlikely to be commissioned if the economic benefits are not captured by the service incurring the implementation costs.^9^

These challenges can be exemplified by considering bioinformatics workflows that identify clinically actionable genetic features,^10^ a process with no current gold standard methods.^11^ Obtaining regulatory approval requires extensive validation, yet bioinformaticians lack experience developing standard operating procedures and performing compliance work. Most jurisdictions require ISO 15189 accreditation, while the USA requires Clinical Laboratory Improvement Amendments or College of American Pathologists accreditation as the gold standard. For most researchers, engaging accredited labs to validate a pipeline is a daunting task. The magnitude of these challenges varies by disease category, with rare disease studies hampered by small cohorts and limited statistical power, while those on common complex conditions must contend with polygenic architectures and large numbers of variants of uncertain causal significance.

Between researchers, clinicians and regulators, there exists an opportunity for the establishment of translational medicine centres. These centres need to operate differently than traditional institutes, using clinical uptake of research discoveries as the measure of success. At the core, such centres require multidisciplinary teams spanning the entire research to clinic continuum: clinician-scientists to evaluate clinical utility, regulatory affairs specialists familiar with national approval pathways (eg, U.S. Food and Drug Administration, European Medicines Agency, Australian Therapeutic Goods Administration), health economists for costing analysis, biostatisticians for clinical trial work and bioinformaticians with experience developing clinical-grade pipelines. Further, experts in health system integration, ethics and data governance are required. Translational medicine centres would differ from existing institutions in that their explicit mandate would be clinical adoption of specific discoveries, through the provision of all necessary regulatory, economic, political and legal support.

The mandate of such centres is end-to-end support starting with early consultations with researchers considering the clinical viability, regulatory pathways, validation data required by regulators and access to ISO 15189 accredited labs. Specifically, this includes steps such as developing standardised measures of performance, designing multicentre validation experiments across diverse patient populations and providing relevant regulatory template documents. Critical to success will be early and consistent engagement of all parties throughout the development process; effective communication channels established from the outset will ensure only the discoveries most likely to succeed go forward.

Beyond personnel, access to computational infrastructure for processing clinical-grade data in a health system compliant manner is needed. Partnerships with pathology services, larger clinical networks and sequencing technology companies are *essential*, with funding models including direct government investment, public-private partnerships or bespoke fee-for-service. Initiatives such as the NIH (National Institutes of Health) Clinical and Translational Science Awards and the Australian Genomics Health Futures Mission provide starting points; however, substantial time and investment is required to deliver impact at scale. The establishment of translational medicine centres will ultimately require individuals to champion the initiative. Coordinated patient advocacy, legislation and the potential for litigation are powerful motivators that, when harnessed effectively, are at least as impactful as political guidelines or economics-driven arguments.^12^

Finally, these centres must explicitly incorporate health equity assessments throughout to prevent new technologies from widening healthcare disparities. Genomic medicine risks entrenching existing inequities, as the benefits of precision medicine have largely accrued to populations of European ancestry in high-income settings, leaving low- and middle-income countries and resource-constrained regional health systems facing a compounding burden of diagnostic delay, misdiagnosis and therapeutic exclusion. This requires evaluating feasibility across a spectrum of healthcare settings considering the training requirements, the scalability of bioinformatics infrastructure and scope for adaptation in resource-limited environments.^13^ Ultimately, success will be measured by the degree to which precision medicine technologies are adopted into routine clinical care and demonstrably improve patient outcomes.

## Data Availability

No data are available.
